# Changes of alpha-fetoprotein levels could predict recurrent hepatocellular carcinoma survival after trans-arterial chemoembolization

**DOI:** 10.18632/oncotarget.20343

**Published:** 2017-08-18

**Authors:** Chao He, Xiaoyun Zhang, Chuan Li, Wei Peng, Tian-Fu Wen, Lv-Nan Yan, Jiayin Yang, Wusheng Lu

**Affiliations:** ^1^ Department of Liver Surgery and Liver Transplantation Center, West China Hospital of Sichuan University, Chengdu 610041, Sichuan Province, China

**Keywords:** recurrent hepatocellular carcinoma, trans-arterial chemoembolization, alpha fetoprotein change, prognosis, hepatectomy

## Abstract

**Background:**

There is paucity of information concerning whether AFP change is a predictor of prognosis for recurrent hepatocellular carcinoma (RHCC) patients after trans-arterial chemoembolization (TACE).

**Methods:**

A total of 177 RHCC patients who received TACE as first-line therapy were retrospectively analyzed. The patients were classified into three groups according to their pre-TACE and post-TACE AFP levels (group A: AFP decreased, group B: AFP consistent normal, and group C: AFP increased). The recurrence to death survival (RTDS) and overall survival (OS) were estimated by the Kaplan-Meier method, and compared by the log-rank test. Multivariate analyses were performed to identify prognostic factors for OS and RTDS.

**Results:**

There was no significant difference among the three groups concerning the baseline characteristics. The median overall survival (OS) was 74.5 months in group A (95% confidence interval (CI): 63.5, 85.6), 64.0 months in group B (95% CI: 52.3, 75.7) and 29.0 months in group C (95% CI: 24.1, 33.9; P<0.001). The median recurrence to death survival (RTDS) was 66.5 months (95% CI: 53.4, 79.6) in group A, 50.4 months (95% CI: 39.5, 61.4) in group B and 17.7 months (95% CI: 13.4, 22.1; P<0.001) in group C. Multivariate analysis revealed that tumor size at resection stage, tumor number at recurrent stage, cycles of TACE, mRECIST response and AFP change after TACE were significant independent risk factors for RTDS and OS.

**Conclusions:**

AFP change could predict the prognoses of patients with RHCC who received trans-arterial chemoembolization, which may help clinicians make subsequent treatment decision.

## INTRODUCTION

Hepatocellular carcinoma (HCC) is the fifth most lethal malignant cancer and the 2nd cause of cancer related death in the world with a still increasing incidence rate [1–3]. China alone accounts for 51% of HCC related death annually worldwide [4]. Hepatectomy has been acknowledged as a curative treatment for hepatocellular carcinoma (HCC). However, recurrence rate after hepatectomy could be 70% at postoperative 5 years [5]. Till now, there is no established guideline addressing the management of recurrent hepatocellular carcinoma (RHCC). Repeated hepatectomy for intrahepatic RHCC is widely recognized as the standard treatment modality. However, only a minority of RHCC patients are amenable, TACE remains the mainstay for unresectable RHCC [6–8]. In order to improve the management of RHCC, it is important to identify prognostic risk factors, especially for RHCC patients who received TACE. Alpha fetoprotein (AFP), a glycoprotein secreted by HCC in approximately 70% of HCC patients, has been routinely used in HCC screening, diagnosis, surveillance, prognostic prediction, as well as in the monitoring of post-treatment HCC recurrence [9–13]. The reduction of AFP is thought to indicate a good response to treatment [9, 14, 15]. However, whether AFP change after TACE can predict prognosis of RHCC has not been investigated before. In the present study, we tried to define a new AFP change criteria, and evaluated its impact on overall survival (OS) and recurrence to death survival (RTDS) of RHCC patients.

## RESULTS

### Clinicopathologic characteristics

**Figure 1:**
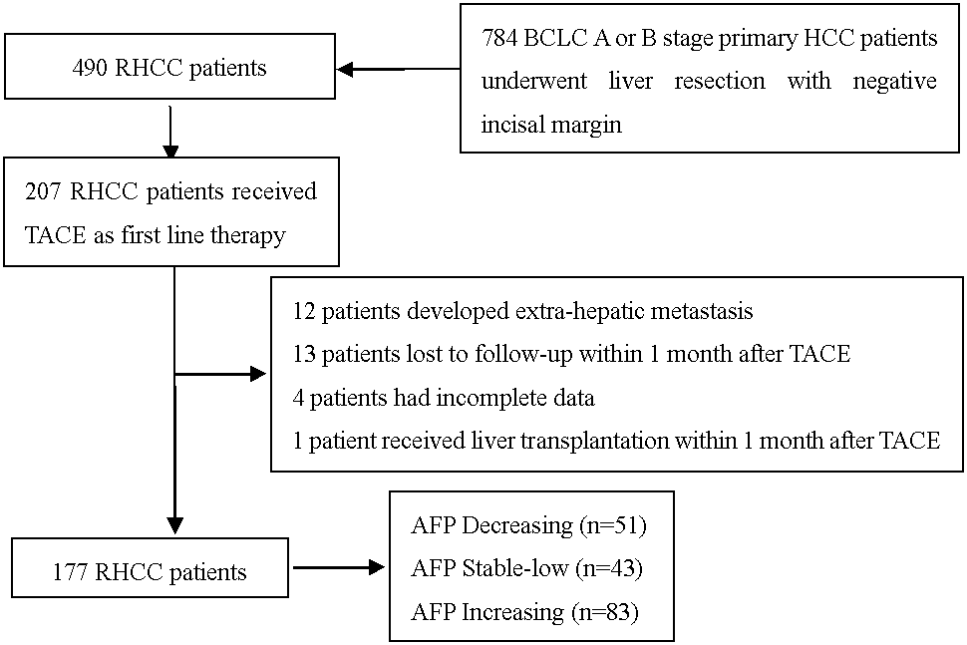
Flowchart of the process for patients’ selection

For the 784 BCLC A or B stage HCC patients who underwent liver resection during our study period, 490 (62.5%) patients developed recurrence. A total of 177 patients were qualified for the study. Details about patient selection were shown in Figure 1. The clinical backgrounds of HCC patients at resection and recurrent stages were provided in Table 1 and Table 2, respectively. There were 152 male (85.8%) and 25 female (14.2%) patients with a median age of 50.5y (range 22.3-79.1y). 155 patients (87.6%) had liver cirrhosis. Elevated AFP (>400 ng/mL) levels were found in 88 cases (49.7%). 54 patients (30.5%) had more than one tumor in the liver. The enrolled patients were classified into three groups: Group A: AFP decreased, Group B: AFP consistent normal; and group C: AFP increased. Significant differences in preoperative AFP level, pre-TACE AFP level and age were observed among the three groups (P < 0.05), whereas no significant difference was observed in gender, tumor number, tumor size, liver cirrhosis, differentiation, MVI, total bilirubin, albumin, CREA, PT, WBC, PLT and recurrence free survival (RFS) among 3 groups at resection stage or recurrence stage. There was no significant difference concerning the number of patients who received subsequent therapy (radiofrequency ablation, liver resection, liver transplantation, chemotherapy, radiotherapy, sorafenib) after recurrence (Table 3).

**Table 1:** Clinical backgrounds of HCC patients at the resection stage (n=177)

Variables	Group A(n=51)	Group B(n=43)	Group C(n=83)	P-value
Age (years)	51.8±13.1	54.4±11.7	46.4±11.7	0.020
Gender, male	40(78.4%)	40(93.0%)	72(86.7%)	0.123
TBIL, umol/L	17.5±16.0	14.2±5.7	14.9±6.1	0.226
ALB, g/L	41.2±4.4	41.4±4.5	41.6±4.4	0.869
PT, s	12.2±1.7	11.7±1.2	15.6±26.8	0.437
WBC,×109/L	5.2±2.1	5.4±1.4	5.6±1.9	0.459
PLT×109/L	127.0±59.5	142.6±64.4	143.7±70.5	0.331
Tumor number				0.283
1	32(62.7%)	33(76.7%)	58(69.9%)	
2-3	15(29.4%)	7(16.3%)	14(16.9%)	
>3	4(7.8%)	3(7.0%)	11(13.3%)	
Tumor size, >5cm	32(62.7%)	25(58.1%)	64(77.1%)	0.056
MVI, positive	15(29.4%)	13(30.2%)	33(39.8%)	0.378
Differentiation, III–IV	16(31.4%)	8(18.6%)	31(37.3%)	0.098
Cirrhosis, presence	73(88.0%)	34(79.1%)	48(94.1%)	0.087
Preoperative AFP, >400 ng/ml	35(68.6%)	5(11.6%)	48(57.8%)	<0.001

TBIL=total bilirubin; ALB=albumin; PT=prothrombin time; WBC=white blood cell; PLT=platelet; MVI= micro-vascular invasion; AFP=α-fetoprotein.

**Table 2:** Clinical backgrounds of HCC patients at the recurrent stage (n=177)

Variables	Group A(n=51)	Group B(n=43)	Group C(n=83)	P-value
Age (years)	51.8±13.1	55.4± 11.8	47.2± 11.9	0.001
Gender, male	40(78.4%)	40(93.0%)	72(86.7%)	0.123
TBIL, umol/L	14.6±6.3	14.5±6.6	16.1±7.2	0.306
ALB, g/L	41.3±4.6	43.1±3.9	41.5±5.5	0.151
ALT, IU/L	48.3±69.3	43.0±27.1	48.0±38.6	0.827
AST, IU/L	47.2±48.3	36.9±15.2	55. 7±79.3	0.256
CREA, mmol/L	74.7±21.2	81.9±24.9	74.6±16.4	0.124
PT, s	12.3±1.3	12.4±4.4	12.4±1.1	0.989
WBC,×109/L	5.1±2.1	5.0±2.0	5.4±1.9	0.452
PLT,×109/L	116.3±58.1	117.4±66.6	126.5±59.8	0.591
Tumor size, >3cm	14(27.5%)	9(20.9%)	30(36.1%)	0.188
Tumor number, n>3	34(66.7%)	30(69.8%)	68(81.9%)	0.102
Pre-TACE AFP, positive	50(98.0%)	0(0.0%)	78(94.0%)	<0.001
RFS, months	11.9±15.6	10.9±15.9	9.5± 10.9	0.600
TACE cycles	3.2±3.0	3.2±2.2	2.4±1.9	0.073

TBIL = total bilirubin; ALB = albumin; ALT = alanine aminotransferase; AST = aspartate aminotransferase; PT = prothrombin time; CREA = creatinine; WBC = white blood cell; PLT = platelet; AFP = α-fetoprotein; RFS = recurrence free survival time; TACE = trans-arterial chemoembolization.

**Table 3:** Subsequent therapies of 177 RHCC patients according to AFP change

Therapy	Group A (n= 51)	Group B (n= 43)	Group C(n=83)	P value
Any/none	16/35	14/29	28/55	0.960
RFA Yes/no	3/48	3/40	5/78	0.972
Resection Yes/no	4/47	5/38	7/76	0.788
Liver transplantation Yes/no	2/49	1/42	5/78	0.620
chemo-, radiotherapy Yes/no	2/49	1/42	2/81	0.854
Sorafenib Yes/no	5/46	4/39	9/74	0.959

### OS according to AFP changes after hepatectomy

**Figure 2:**
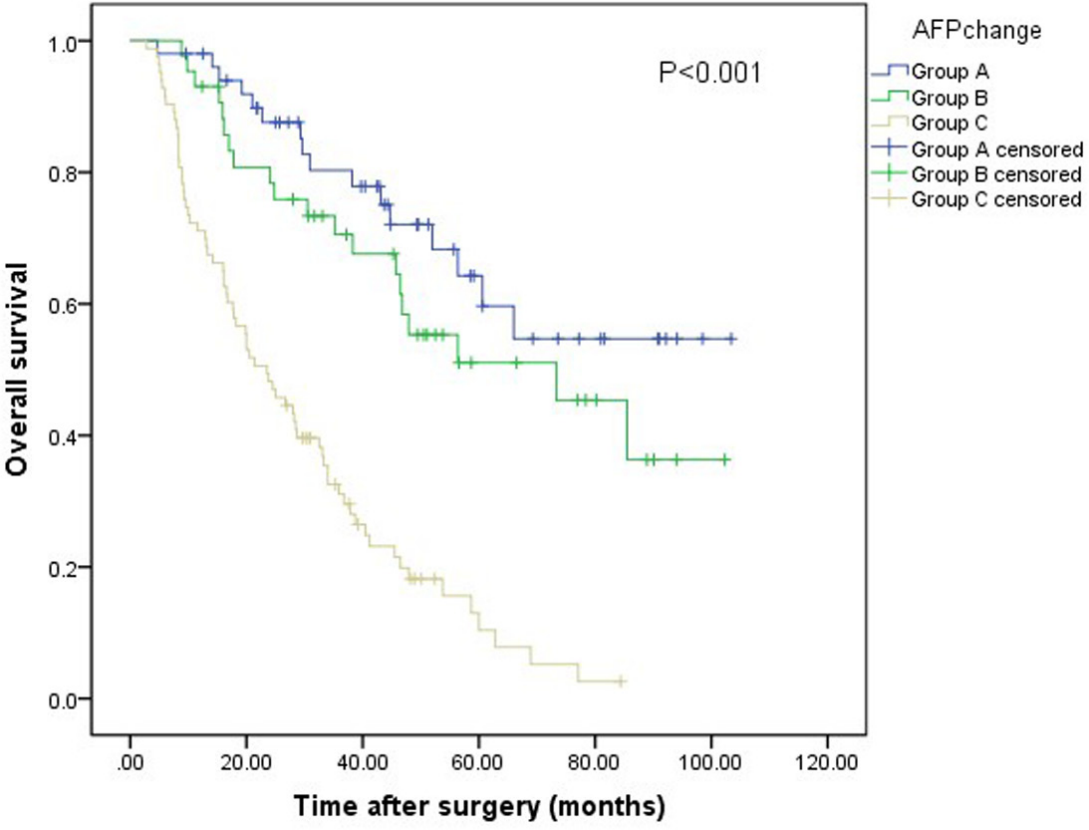
Relationship between AFP changes and OS of patients with RHCC after liver resection The difference of OS among the three groups was significant (log-rank test, P<0.001).

The 1-, 3-, and 5-y OS rates of the entire series were 84.2%, 54.4%, and 35.5%, respectively. The OS rates were compared among the three groups of patients. The median overall survival (OS) was 74.5 months in group A (95% confidence interval (CI): 63.5, 85.6), 64.0 months in group B (95% CI: 52.3, 75.7) and 29.0 months in group C (95% CI: 24.1, 33.9). The 1-y OS rates of groups A, B, and C were 98.0%, 93.0%, and 71.1%, respectively, and the 3-y OS rates were 80.3%, 70.5%, and 31.1%. The 5-y OS rates of groups A, B, and C were 64.3%, 51.1% and 10.4%. P<0.001 (Figure 2).

### RTDS according to AFP changes after recurrence

**Figure 3:**
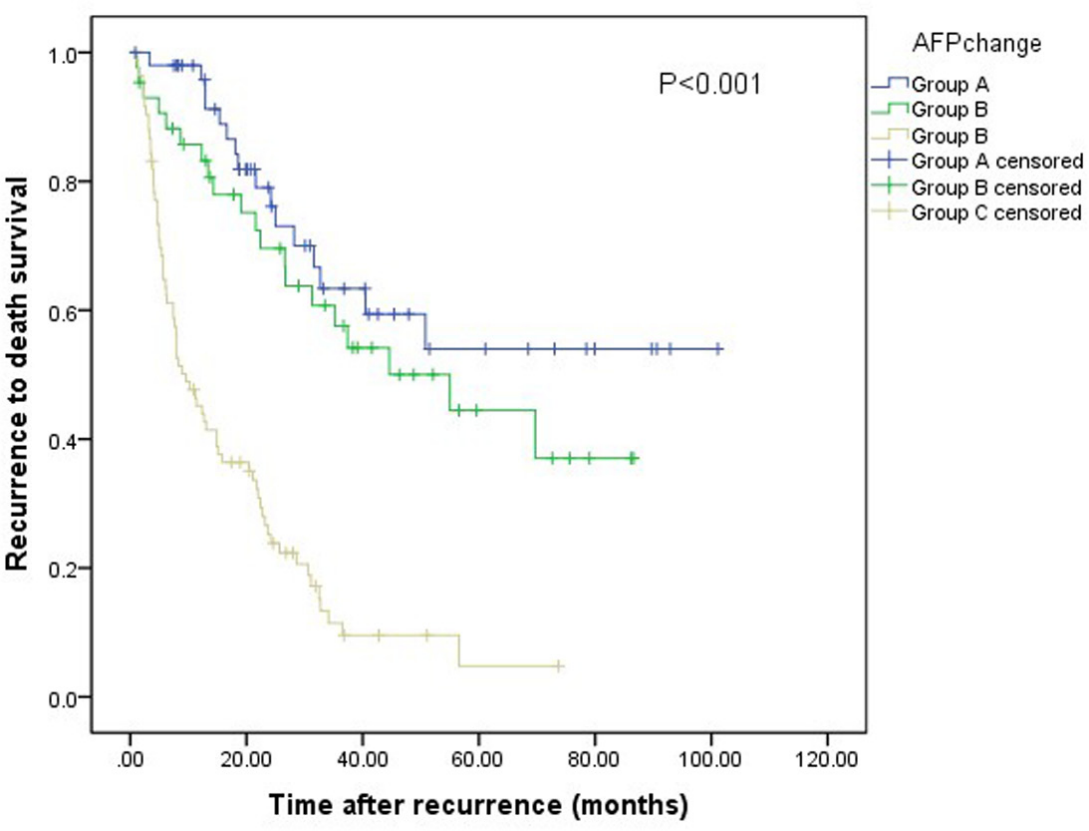
Relationship between AFP changes and RTDS of patients with RHCC after TACE The difference of RTDS among the three groups was significant (log-rank test, P<0.001).

The 1-, 3-, and 5-y RTDS rates of the entire series were 69.9%, 37.1%, and 28.5%, respectively. The median survival time after recurrence was 66.5 months (95% CI: 53.4, 79.6) in group A, 50.4 months (95% CI: 39.5, 61.4) in group B and 17.7 months in group C (95% CI: 13.4, 22.1). The RTDS rate was higher in group A than in group B and C (98.0%, 85.7% and 45.2% at 1 y and 63.4%, 57.6% and 11.5% at 3 y, 54.0%, 44.4% and 4.8% at 5 y respectively, P<0.001 (Figure 3).

### Intrahepatic lesions response

During the last follow-up, two experienced board-certified radiologists evaluated the intrahepatic lesions responses by using CT or MRI according to the mRECIST assessment of HCC [16]. Tumor response was assessed at 1 month after TACE. The intrahepatic lesions control rate was calculated with the following equation: DCR = (SD + PR + CR)/N, where SD is number of patients with stable disease, PR is number of patients with partial response, and CR is number of patients with complete response. The intrahepatic lesions control rate was 98.0% for group A, 55.8% for group B and 25.3% for group C. The difference was significant among the three groups (P<0.001, Table 4).

**Table 4:** Intrahepatic lesions responses for the three groups according to mRECIST criteria

Intrahepatic lesions responses	Group A	Group B	Group C	P value
Total patients	51	43	83	
Complete response	5	1	0	
Partial response	39	18	7	
Stable disease	6	5	14	
Progressive disease	1	19	62	
Disease control rate (%)	98.0	55.8	25.3	<0.001

Disease control rate (DCR) = (CR + PR+ SD)/N. PR=partial response. CR=complete response. SD= stable disease, N=177.

### Independent predictors of RTDS

The results of the univariate and multivariate analyses of the RTDS were illustrated in Table 5. Univariate and multivariate analyses showed that tumor size at resection stage (HR: 1.910, 95% CI: 1.180-3.092, P =0.008), tumor number at recurrent stage (HR: 4.004, 95% CI: 2.040-7.856, P<0.001), AFP change after TACE (HR: 1.650, 95% CI: 1.174-2.319, P=0.004), cycles of TACE (HR: 0.899, 95% CI: 0.821-0.985, P=0.022), mRECIST response (HR: 0.539, 95% CI 0.319-0.912, P=0.021) were independent predictors for RTDS.

**Table 5:** Multivariate analysis of the risk factors for RTDS

Variables	Univariate	Multivariate analysis		
	P value	Hazard ratio	95%CI	P value
Resection stage				
Gender (male vs female)	0.669			
Tumor size (>5 vs ≤5cm)	0.028	1.910	1.180-3.092	0.008
Tumor number (1, 2-3, >3)	0.504			
MVI (A vs P)	0.621			
Liver cirrhosis (A vs P)	0.344			
Differentiation (W, M, L)	0.744			
Preoperative AFP (≥400 versus <400 ng/ml)	0.541			
Recurrent stage				
TBIL	0.134			
ALT	0.681			
AST	0.507			
ALB	0.731			
CREA	0.915			
PT, s	0.433			
WBC	0.614			
PLT	0.662			
Tumor size (>3versus ≤3cm)	0.531			
Tumor number(>3 versus ≤3)	<0.001	4.004	2.040-7.856	<0.001
Pre-TACE AFP (>8 vs ≤8 ng/ml)	0.541			
RFS	0.447			
AFP change	<0.001	1.650	1.174-2.319	0.004
Cycles of TACE	<0.001	0.899	0.821-0.985	0.022
mRECIST, DC vs PD	<0.001	0.539	0.319-0.912	0.021

AFP=α-fetoprotein; A=absent; P=present; L=low differentiation; M=moderate differentiation; W =well differentiation; MVI= microvascular invasion; TBIL=total bilirubin; ALT=alanine aminotransferase; AST= aspartate aminotransferase; ALB=albumin; PT=prothrombin time; WBC=white blood cell; PLT=platelet; CI=confidence interval; RFS =recurrence free survival time; TACE=trans-arterial chemoembolization; DC=disease control; PD=disease progression.

### Independent predictors for OS

The results of the univariate and multivariate analyses of the OS were illustrated in Table 6. Univariate and multivariate analyses demonstrated that tumor size at resection stage (HR: 1.949, 95% CI: 1.221-3.113, P=0.005), tumor number at recurrent stage (HR: 3.650, 95% CI: 1.869-7.127, P<0.001), AFP change after TACE (HR: 1.668, 95% CI: 1.204-2.311, P=0.002), cycles of TACE (HR: 0.896, 95% CI: 0.822-0.976, P=0.012), mRECIST response (HR: 0.540, 95% CI: 0.333-0.876, P=0.012) were independent predictors for OS.

**Table 6:** Multivariate analysis of the risk factors for OS

Variables	Univariate	Multivariate analysis		
	P value	Hazard ratio	95%CI	P value
Resection stage				
Gender (male vs female)	0.669			
Tumor size (>5 vs ≤5cm)	0.028	1.949	1.221-3.113	0.005
Tumor number (1, 2-3, >3)	0.504			
MVI (A vs P)	0.621			
Liver cirrhosis (A vs P)	0.344			
Differentiation (W, M, L)	0.744			
Preoperative AFP (≥400 versus <400 ng/ml)	0.541			
Recurrent stage				
TBIL	0.134			
ALB	0.731			
ALT	0.681			
AST	0.507			
PT, s	0.433			
WBC	0.614			
CREA	0.915			
PLT	0.662			
Tumor size (>3versus ≤3cm)	0.531			
Tumor number (>3 versus ≤3)	<0.001	3.650	1.869-7.127	<0.001
Pre-TACE AFP (>8 vs ≤8 ng/ml)	0.541			
RFS	0.447			
AFP change	<0.001	1.668	1.204-2.311	0.002
Cycles of TACE	<0.001	0.896	0.822-0.976	0.012
mRECIST, DC vs PD	<0.001	0.540	0.333-0.876	0.012

AFP=α-fetoprotein; A=absent; P=present; L=low differentiation; M=moderate differentiation; W =well differentiation; MVI= microvascular invasion; TBIL=total bilirubin; ALT=alanine aminotransferase; AST= aspartate aminotransferase; ALB=albumin; PT=prothrombin time; WBC=white blood cell; PLT=platelet; CI=confidence interval; RFS =recurrence free survival time; TACE=trans-arterial chemoembolization; DC=disease control; PD=disease progression.

## DISCUSSION

The treatment of RHCC remains controversial. Treatment strategies for intrahepatic RHCC include salvage liver transplantation, re-resection, trans-arterial chemoembolization (TACE), and ablative therapies. Liver transplantation and re-resection are widely acknowledged as the most effective treatment modality [6, 17, 18]. However, liver transplantation is largely restricted by the donor shortage. Re-resection is comprised by reduced liver volume, presence of liver cirrhosis, the multifocal nature, location of the tumor and high recurrence rate. The re-resection rate for RHCC has been reported to be only 10% to 31% [6, 7]. Ablative therapies with percutaneous ethanol injection (PEI) or radiofrequency ablation (RFA), has been recognized as curative treatment in primary HCC. It is only recommended to small HCC. TACE is a widely used minimal invasive therapy with low mortality and morbidity rates and less application restriction than liver transplantation, resection and ablative therapy [19]. Previous researches have shown that TACE can provide similar benefit result to ablative therapies [20–22]. However, there are diverse outcomes after TACE in terms of treatment response and survival. Therefore, to further improve the management of RHCC, it is crucial to identify the risk factors associated with long-term survival after TACE.

In 2005, Chen et al. first introduced the concept of ‘AFP response’. AFP response was defined as a 50% or greater reduction of AFP levels for 4 or more weeks during treatment. They reported that serum AFP response was independent prognostic factor for both progression-free survival and overall survival, whereas radiographic response was not [23]. Subsequently, this finding was further validated in other modalities of treatments (such as TACE, sorafenib, RFA). The baseline AFP levels were different in these studies, either higher than 200 ng/mL, 100 ng/ml, or 20 ng/ml. Other definitions of AFP response or AFP change were applied with different cut-offs of the reduction of AFP after treatment. For example, AFP response or change were defined as AFP reduction greater than 20%, 46% and 50% [24–28]. Therefore, a universal definition of AFP response or AFP change to predict the prognosis after treatment for various treatment modalities and different tumor stage may be impossible. Although AFP change after therapies has been proved to correlate the survival of primary HCC patients. Little is known about its role in RHCC. The AFP status of RHCC is unique, as it is not always consistent with its initial stage [29]. Although the AFP of resection stage is normal, it could be abnormal, even high, and vice versa. In the present study, we did not designate a certain baseline AFP level like previous studies, but analyzed all the RHCC patients who received TACE instead, regardless of their baseline AFP. We used a novel criteria of AFP change and compared the prognosis of three groups, which was significant different from previous researches. We found for the first time that the AFP change could predict prognosis of RHCC patients who received TACE. In addition, our study demonstrated that tumor size at resection stage, tumor number at recurrent stage were also prognostic factors of survival. These findings are consistent with previous results [29]. However, in contrast to previous findings, the present study suggest that pre-operative and pre-TACE elevated AFP were not a predictor of prognosis [30].

In the present study, we defined AFP decreased as baseline AFP abnormal (>8 ng/ml) and AFP change <0 after TACE, AFP increased as post-TACE AFP >8 ng/ml and AFP change ≥0, and AFP consist normal as both the pre-TACE AFP and post-TACE are normal. This definition may be better to predict survival of the RHCC patients who received TACE than previous definitions. First, AFP positivity only appear in approximately 70% of HCC patients. In AFP normal HCC patients, the previous AFP response criteria for predicting prognosis may not be applicable. Second, AFP rapidly decline to normal range after radical eradication of cancer cells and rise again after recurrence. With regular and more frequent follow up, RHCCs are detected earlier than primary HCC. Patients with baseline AFP>200 ng/ml or AFP>20 ng/ml were only seen in a proportion of HCC patients. Therefore, previous AFP response criteria was largely restricted for its application in RHCC patients. In the present study, we found that AFP increased group had the least survival after TACE while the AFP decreased group had the best survival after TACE, and the AFP consistent normal group had the medium survival, which has not been reported before.

Hypoxia and tumor necrosis after therapy has been shown to lead to AFP decrease after treatment [24, 31]. Serum AFP increase was associated with HCC progression [32], while AFP decrease may predict advantageous therapeutic effect. AFP is secreted by HCC, but also participate in the pathogenesis of HCC. There are several mechanisms, including AFP promoting HCC growth, proliferation, metastasis, preventing apoptosis and escaping from immune surveillance [33–37]. And AFP has also been reported to be a predictor of MVI [38]. Therefore, AFP may predict the aggressivity of HCC, and which is plausible for AFP increased group and AFP decreased group to have opposed survival. Our study also demonstrated that the prognosis of AFP consistent normal group was superior to the AFP increased group but inferior to the AFP decreased group. Possible explanations were that although AFP has also been shown to be a risk factor which contribute to mortality and morbidity of HCC and AFP negative HCCs have been reported to have superior prognosis than AFP positive HCCs, the AFP consistent normal HCCs were a mixture of HCCs which did not secret AFP, and AFP secreting HCCs which AFP were secreted at a low level by the time of recurrence detection, and were also a mixture of HCCs of well and bad responses to TACE.

In the present study, we also found that cycles of TACE was also an independent prognostic factor to long term survival for RHCC patients who received TACE as first-line therapy. As TACE was not a curative therapy for HCC, and according to a systematic review, the objective response rate, defined as sum of complete and partial response, was only 52.5% [39]. Therefore, in order to enhance the efficacy of TACE, subsequent therapies are paramount. If liver transplantation, re-resection and ablative therapy are not feasible after TACE, repeated TACE would be an important method to improve survival.

Our study has some limitations. First, the study was a retrospective study carried out in a single center, and the sample size was relatively small. Second, in the present study, we recruited BCLC A and BCLC B HCC patients and excluded BCLC C stage HCC patients as eligible BCLC-C stage patients were quite few in our hospital and also by which it would be more convenient to control confounding factors. Therefore, whether AFP change can also predict the prognosis of BCLC C stage HCC patients still needs further studies. Third, other risk factors such as HBV-DNA level, tumor capsular invasion, satellite nodules, were not analyzed in the present study. Forth, the follow-up was relatively short.

In conclusion, AFP change was found to be a reliable predictor for RHCC patients who received TACE as first-line treatment. AFP change is a simple, rapid, reproducible, and operator-independent measurement and AFP monitoring should be included as part of regular follow-ups in HCC patients after surgery and TACE, particularly for AFP-positive patients. It may be beneficial for clinicians to detect early recurrence and would help clinicians make subsequent treatment decision. RHCC patients with increased AFP levels and progression disease according to image after TACE should receive additive therapies. Repeated TACE is useful in improving long term survival when liver transplantation, re-resection and ablative therapy are not feasible for RHCC patients after TACE treatment.

## MATERIALS AND METHODS

### Patients

From Oct 2007 to May 2016, 784 BCLC A or B stage primary HCC patients underwent liver resection for HCC at the West China Hospital of Sichuan University. Those patients who developed RHCC were identified from our prospectively collected database. These patients with RHCC who had TACE as first line therapy were further identified for the study.

Patients who met the following criteria were enrolled: (1) HCC who received curative resection for primary tumor as described in our previous report; (2) without macro-vascular invasion and extra-hepatic metastasis; (3) pathologic report revealed negative incisal edge; (4) regularly followed-up with AFP and imaging. Excluding criteria include: (1) Patients with incomplete clinical data; (2) lost to follow-up within 1 month after TACE; (3) recurrence with extra-hepatic metastasis; (4) received therapies other than TACE as first line therapy; (5) received RFA, hepatectomy and liver transplantation within 1 month after TACE.

Based on the above criteria, 30 patients were excluded. Excluded patients were as follows: 12 patients developed extra-hepatic metastasis, 13 patients lost to follow-up in 1 month after TACE, 4 patients had incomplete data, 1 patient received liver transplantation within 1 month after TACE. Finally, a total of 177 patients were enrolled into this study. Clinical variables, including demographic data, complete blood counts, liver function tests, AFP, and tumor features, including tumor number, MVI, tumor differentiation, and maximum diameter, cirrhosis background, were prospectively collected and retrospectively reviewed. Diagnoses of HCC was confirmed by postoperative histopathologic examination. Microvascular invasion (MVI) was identified under a light microscope by pathologists. HCC was histologically classified using the Edmondson–Steiner classification. The study was approved by the Ethics Committee of the West China Hospital of Sichuan University.

### Definition of AFP change

All pre-TACE AFP levels were taken within 5 days before TACE. Post-TACE AFP was taken in the first follow-up visit (1 month after TACE). AFP change was calculated by post-TACE AFP minus pre-TACE AFP. If the baseline pre-TACE AFP was positive (>8 ng/ml), and the AFP change was <0, it was defined as AFP decreased, if post-TACE AFP was positive (>8 ng/ml), and the AFP change ≥0, it was defined as AFP increased. And if the pre-TACE AFP and post-TACE AFP were both negative (<8 ng/ml), it was defined as AFP consistent normal.

### TACE

The Seldinger technique was used for the application of TACE. Procedure was as follows: after local anesthesia, the femoral artery was punctured, a catheter (Microferret; Cool, Bloomington, IN, USA) was inserted through the femoral artery and advanced toward the tumor-feeding arteries with the help of a guidewire for selective embolization, and TACE of the feeding arteries was performed through further super-selective catheterization as close to the tumor as possible. A mixture of doxorubicin hydrochloride (Adriamycin; Ildong Co., Ltd., Seoul, Korea) and an emulsion of iodized oil (Lipiodol; Laboratorie Guerbet, Aulnay Sous Bois, France) was used for chemoembolization. The dose of the embolization agent was determined according to the tumor size, tumor number, feeding vessels and liver function status. After embolization, angiography was performed to determine the extent of vascular occlusion and to assess the blood flow in other arterial vessels. After the procedure, patients were requested to put pressure on the entry site for half an hour to ensure hemostasis. About 1 month after TACE, an abdominal-enhanced CT was performed, and serum AFP level was measured to determine if a subsequent application of TACE was necessary and whether other treatment modalities, such as liver resection, RFA, liver transplantation, were feasible.

### Follow-up

After liver resection, all patients were followed up at the first, third, and sixth months in the first half year after the operation, every 3 months during the subsequent 3 year, and every 6 months thereafter. Antiviral drugs such as nucleoside acid analogs were administered to the patients with positive HBV-DNA tests before or after hepatectomy. Blood cell tests, liver function tests, AFP measurements, HBV-DNA tests, and visceral ultrasonography or computed tomography or magnetic resonance imaging, and chest radiography were performed in the follow-up examinations. Bone scintigraphy was performed whenever HCC recurrence was suspected. PET/CT was performed when RHCC was suspected but contrast-enhanced abdominal ultrasound or CT or MRI, chest image and bone scintigraphy were negative. The diagnosis of intrahepatic RHCC was made when space occupying lesion was found by contrast-enhanced CT or MRI with typical wash-in/wash-out image feature. The overall survival (OS) time was defined as the interval between the operation and death or the last follow-up. The recurrence to death time (RTDS) was defined as the interval between HCC recurrence and death or the last follow-up. The median follow-up was 41.2 months, and the range was 2.8-103.4 months. The last follow-up date was the end of December 2016.

### Statistical analysis

The continuous variables were expressed as the mean ± the standard deviation. The categorical variables were presented as numbers (percentages). The categorical variables were compared with chi-square or Fisher’s exact tests, and the continuous variables were compared with one-way ANOVA for normal distributed data and Kruskal-wallis H test for abnormal distributed data. Survival analysis was performed by using the Kaplane-Meier method, and differences of OS and RTDS among the three groups were compared with the log-rank test. Independent risk factors for RTDS and OS were identified with a Cox regression model using a forward stepwise method. All statistical analyses were performed with the SPSS 20.0 statistical software (Chicago, IL, USA). Calculated P values were two-sided, and a P value <0.05 was considered statistically significant.

## Abbreviations

TBIL: total bilirubin
ALB: albumin
ALT: alanine aminotransferase
AST: aspartate aminotransferase
PT: prothrombin time
CREA: creatinine
WBC: white blood cell
PLT: platelet
MVI: microvascular invasion
AFP: α-fetoprotein
TACE: trans-arterial chemoembolization
OS: overall survival
RFS: recurrence free survival
RTDS: recurrence to death survival
CI: confidence interval
